# Manufacturability of a Tetraethyl Orthosilicate-Based Hydrogel for Use as a Single Application Otitis Externa Therapeutic

**DOI:** 10.3390/pharmaceutics14102020

**Published:** 2022-09-23

**Authors:** Emma Barrett-Catton, Elizabeth M. Arrigali, Bogdan A. Serban, Kolton C. Sandau, Monica A. Serban

**Affiliations:** 1 Department of Biomedical and Pharmaceutical Sciences, University of Montana, Missoula, MT 59812, USA; 2 Montana Biotechnology Center (BIOTECH), University of Montana, Missoula, MT 59812, USA

**Keywords:** tetraethyl orthosilicate, hydrogel, nanoparticles, drug release, hyaluronan, manufacturing, otitis externa

## Abstract

Otitis externa, also known as outer ear infection, is a frequent affliction in both humans and animals. The most prevalent treatment for otitis externa is ear drops, but it is difficult to adhere properly to this treatment, causing poor patient compliance and the potential for complications. As a result, we have developed a tetraethyl orthosilicate-based hydrogel for use as single application treatment for otitis externa to increase ease of use and improve patient outcomes. Herein, we investigated the manufacturability of the hydrogel, focusing on several key aspects: formulation repeatability and reproducibility, material source and tolerances, release of a variety of model drugs, and impact of application-specific physiological factors, specifically local pH and enzymatic activity on drug release. Overall, our results indicate that these hydrogels are well suited for production and scalability, as they have a robust manufacturing process, have a wide tolerance for pH level, release a variety of model drugs, and are not impacted by outer ear canal-specific physiological factors.

## 1. Introduction

Otitis externa, or outer ear infection, is a common ailment that impacts approximately 1% of the human population yearly, and 10% of the human population over the course of their lifetime [1,2]. It is most commonly caused by bacteria, specifically *Pseudomonas aeruginosa* and *Staphylococcus aureus* [2]. Typically, otitis externa is treated by cleaning the ear canal and then treating the infections with topical medications, or oral medications if the infection has spread beyond the ear [1,2]. Topical medications are preferred over oral medications, as they directly target the infected tissue, and oral medications have higher risk of developing drug resistant bacteria [2,3,4,5]. However, there is poor patient compliance with topical treatments, as they typically require multiple treatments a day for 7–10 days, with one study finding that only 40% of patients properly complied with the topical treatment over the course of the just the first 3 days [3,4,5]. Poor compliance with the antibiotic regime can lead to continued or worsening infection, which can be especially detrimental for elderly and diabetic populations who are at increased risk for malignant otitis externa, a potentially deadly condition [1,2,5,6,7].

Otitis externa is also a very prevalent issue in animal medicine, occurring at a rate of 5–20% in dogs and about 2% in cats [8,9,10,11]. It is associated with many different pathogens, including the bacteria *Staphylococcus intermedius* and the yeast *Malassezia pachydermatis*, along with various hypersensitivities and diseases [8,9,10,11]. Topical treatments are also commonly used to treat canine otitis externa, which require multiple drops once or twice a day [10,12]. This treatment is difficult to adhere to with animals, with one study finding that only 10% of owners were able to apply the correct number of ear drops to their pet during the course of the treatment, which, similar to human cases, can lead to continued or worsening infection [13].

As a result of the common issue of poor patient compliance in both human and veterinary treatment, there is a clear need for a single application topical treatment for otitis externa, eliminating the issue of patient compliance and thereby improving patient outcomes. There is currently one FDA approved single-application treatment for otitis externa, a ciprofloxacin otic suspension [14,15]. However, the ciprofloxacin otic suspension is cold-chain dependent, and the preparation involves multiple steps that increase the likelihood of user error [14,16]. Another treatment that has been studied is a silver nitrate-based gel [17], but this gel can cause further complications for patients with metal allergies [18]. There are also currently several single application treatments for otitis externa available in veterinary medicine [9,10]. However, both the current single application treatments available for veterinary applications have flaws that reduce their efficacy due to their lack of compatibility with animal physiology and/or behavior. One of the treatments available is applied as a gel, which can make it difficult to reach the site of infection as dog ears are L-shaped [9,19]. Another leading formulation is available as a liquid, which may be mostly ejected from the ear when the animal shakes its head after application [10,19].

In response to the issue of patient compliance with ear drops and the flaws with the alternative single application treatments, we have developed a single application thixotropic hydrogel for antibiotic delivery made of hydrolyzed tetraethyl orthosilicate (hTEOS) and sodium hyaluronate (HA), a polymeric glycosaminoglycan with intrinsic anti-inflammatory properties, for treatment of otitis externa [16,20,21,22,23]. Our gel is cold-chain independent and consists of an easy preparation to minimize user error [16]. Additionally, the thixotropic nature of the gel is advantageous because the hydrogel liquifies when put under stress (e.g., when pushed through a syringe or other type of deployment device nozzle). This allows for easy application as a liquid that coats the entire ear canal, and subsequently rapidly reforms as a gel that deploys its therapeutic cargo in situ [16,20,21]. We have previously comprehensively characterized and demonstrated the utility of these gels for the purpose of a single application treatment of otitis externa [16,21]. The scope of the current study is the assessment of general product development considerations, and of the manufacturability of these hydrogels through evaluation of materials, release of model drugs, and impact of common outer ear canal-specific physiological factors on overall release profiles. The thixogel formulation used herein was selected based on our previous findings on overall material characteristics [16,21].

## 2. Materials and Methods

### 2.1. Materials

Tetraethyl orthosilicate (TEOS) was purchased from Acros Organics (TEOS A, Geel, Belgium) and Alfa Aesar (TEOS B, Lancashire, UK), both now Thermo Scientific (Waltham, MA, USA). Sodium Hyaluronan (HA, 5 kDa) was from Lifecore Biomedical (HA A, Chaska, MN, USA) and HTL Biomedical (HA B, Javené, France). Acetic acid (HOAc) was from EMD Millipore (Billerica, MA, USA), ammonium hydroxide (NH_4_OH) was from Fisher Chemical (Fair Lawn, NJ, USA), and phosphate-buffered saline (PBS) was from Corning Life Sciences (Durham, NC, USA). Fluorescein was from Fluka Analytical, owned by Sigma Aldrich (St. Louis, MO, USA), fluorescein disodium was from Alfa Aesar, now Thermo Scientific (Waltham, MA, USA), green fluorescent protein (GFP) was from Novus Biologicals (Littleton, CO, USA), and blue dextran with molecular weights of 5597, 15,038, and 500,000 g/mol, referred to as 5000, 20,000, and 500,000 g/mol, respectively, was from Sigma Aldrich (St. Louis, MO, USA). Skin Irritation Test kit (SPI-200-SIT) was from MatTek (Ashland, MA, USA), thiazolyl blue tetrazolium bromide (MTT) was from Thermo Scientific (Waltham, MA, USA), and isopropanol was from VWR (Radnor, PA, USA). Hyaluronidase was from MP Biomedicals (Solon, OH, USA), and hydrochloric acid (HCl) was from Janssen Pharmaceuticals (Beerse, Belgium).

### 2.2. Hydrogel Formation

TEOS was activated via hydrolysis (hTEOS) with 0.15 M HOAc for 1.5 h at a 1:9 *v*/*v* ratio. hTEOS was then either combined with aqueous HA (10% *w*/*v* or 5% *w*/*v*) or 1x PBS at a 1:2 *v*/*v* ratio. HA at 10% *w*/*v* was used in the experiments to formulate the gels unless otherwise indicated. The mixtures were vortexed, and the pH was adjusted to ∼7.65 with 1.5 N NH_4_OH. Additionally, gels adjusted to pH levels of 7.3 and 8.0 were also tested. Gels formed when mixtures were left unstirred overnight at room temperature.

### 2.3. Drug Release Studies

Fluorescein, fluorescein disodium, GFP, and blue dextran were used as model drugs because of their variable properties and ease of monitoring. Blue dextran was used at three different molecular weights: 5000, 20,000, and 500,000 g/mol. A fluorescein stock solution of 10 mg/mL was prepared using 0.15 M NH_4_OH, a fluorescein disodium stock solution of 10 mg/mL was prepared using PBS, and a GFP stock solution of 1 mg/mL was prepared using PBS. For the fluorescein, fluorescein disodium, and GFP release, hydrogel aliquots prior to gelation (1980 µL) were transferred to 4 mL glass vials containing 20 µL of stock solution, to yield a total volume of 2 mL containing 100 µg/mL fluorescein or fluorescein disodium or 10 µg/mL GFP. For the blue dextran release, blue dextran in powdered form was added to the HA solutions prior to mixing with hTEOS and 1.5 N NH_4_OH. The solutions were aliquoted into 4 mL glass vials with 2 mL of hydrogel in each. Each blue dextran gel contained 3 mg/mL of blue dextran. For all the model drugs, the gel mixtures containing the dyes were left overnight at room temperature to form gels and subsequently washed with PBS. After washing, 2 mL of PBS, or 0.5 mL of PBS for GFP release, were added to each vial. The vials were then placed at 37 °C with no shaking. The release was monitored at 24 h intervals by assaying 100 µL PBS in duplicate from each vial. The PBS was discarded and replaced with fresh aliquots daily, for each vial. This experimental design was focused primarily on the assessment of the impact of hydrogel formulation and drug loading mechanism on the release of different drugs and not intended to mirror drug release rates under pathological in situ conditions [24]. The drug release was measured using a Cytation 5 Imaging Multi-Mode Reader (BioTek, Winooski, VT, USA). For the fluorescein and fluorescein disodium studies, drug release was monitored by recording the absorbance at 450 nm. For the GFP studies, drug release was monitored by recording fluorescence with an excitation of 488 nm and an emission of 507 nm. For the blue dextran studies, drug release was monitored by recording the absorbance at 380 nm. For both the blue dextran and GFP studies, a blank gel without drug was run alongside the gels with drug loaded, and the ‘release’ from the blank gel was subtracted from the release from the gels containing the model drug, as there is a component released by the gels that absorbs and has fluorescence at the same wavelengths used to measure blue dextran and GFP concentrations.

Two alternative loading strategies were tested for the release of fluorescein and fluorescein disodium. For loading strategy 1, the hTEOS was made and kept for 7 days at room temperature. Then, gels were made for release in the same manner as above. For loading strategy 2, hTEOS was made and mixed with fluorescein or fluorescein disodium at 1 µg/mL concentration and then kept at room temperature for 7 days. After 7 days, the gels were made by mixing one part of the hTEOS and dye mixture and two parts 10% HA and adjusting the pH to approximately 7.65 using 1.5 M NH_4_OH. Both sets of gels were stored overnight under normal environmental conditions to ensure complete gelation, and then the same wash and release protocol was used as outlined above. For loading strategy 1, the fluorescein or fluorescein disodium release was measured using the same method as above. For loading strategy 2, the fluorescein or fluorescein disodium release was measured using fluorescence with an excitation of 485 nm and an emission of 528 nm using the Cytation 5 Imaging Multi-Mode Reader (BioTek, Winooski, VT, USA).

For drug release in different hyaluronidase concentrations and pH levels, the PBS added to the top of the gels was modified to contain hyaluronidase or have different pH levels. For the hyaluronidase release, the PBS was modified to contain 100, 10, 1, and 0 U/mL of hyaluronidase, using hyaluronidase with 749 U/mg. The hyaluronidase release gels were incubated at 37 °C with gentle agitation (50 rpm) in a New Brunswick Scientific Co. Classic C24 incubator shaker (Eppendorf, Hamburg, Germany). The wash, release, and monitoring protocol from above was replicated for these studies, using PBS with hyaluronidase in place of PBS for all the steps. For the pH release, the PBS was modified to have a pH of 4.0 using HCl and compared to a usual run where the PBS had a pH of 7.6. The wash, release, and monitoring protocol from above was replicated for these studies, using PBS at a pH of 4.0 in place of PBS for all steps.

### 2.4. Rheological Characterization

Rheological data was obtained using a hybrid Discovery HR-2 Rheometer/Dynamic Mechanical Analyzer (TA Instruments, New Castle, DE, USA). All hydrogels were characterized within the materials’ pseudolinear viscoelastic range with a 1.00 mm gap, at room temperature. A conditioning step was performed at the start of each test to ensure an axial force between 0.1 and 0.3 N. Oscillatory strain sweeps for thixotropy investigation were conducted with a 20 mm parallel plate geometry within a strain range of 1–100% and an angular frequency of 10 rad/s. The gels went through 3 cycles with a 30 s rest between each cycle. Oscillatory frequency sweeps from 100 Hz to 0.1 Hz with stress of 50 Pa were performed to classify the storage modulus of the gels.

### 2.5. Dry Substance Determination

The dry substance of the gels was determined using an MJ33 moisture analyzer (Mettler Toledo, Columbus, OH, USA). A sample of gel was placed in the analyzer and spread out evenly. The gel was then dried at 104 °C until the weight stopped decreasing. The dry substance percentage was calculated by dividing the final weight by the initial weight before drying.

### 2.6. Skin Irritation Tests

A MatTek In Vitro Epiderm Skin Irritation test, containing 24 tissues, was used to evaluate the hydrogels. In brief, after receiving the tissues, they were taken off the agarose and incubated overnight in the assay media at 37 °C and 5% CO_2_. The tissues were then dosed for one hour with 30 µL gel solution, either a 5% HA, or 10% HA gel, made immediately prior to dosing by adding enough 1.5 N NH_4_OH to induce gelation of a solution containing a one-part hTEOS and two parts either 5% *w*/*v* HA or 10% *w*/*v* HA. PBS was used as a negative control and a 5% SDS solution was used as a positive control. Six tissues were dosed with each gel, and three tissues were dosed with each control. After 60 min, tissues were washed with PBS, placed into fresh media, and incubated for 24 h at 37 °C and 5% CO_2_. After 24 h, the media was changed, and the tissues were incubated again overnight. The tissues were then placed in 1 mg/mL MTT and incubated at 37 °C and 5% CO_2_ for 3 h, then the MTT was removed, and the tissues were washed with PBS. The tissues were then covered with isopropanol, and the plate was sealed with parafilm. The plate was placed in a New Brunswick Scientific Co. Classic C24 incubator shaker (Eppendorf, Hamburg, Germany) at room temperature, shaking at 120 rpm for three hours. Two 200 µL aliquots were taken from each well, and the absorbance was read at 570 nm. The relative viability of each tissue was calculated using the following equation:

relative viability = [absorbance of the tissue ⁄ mean absorbance of the negative control] × 100(1)

where a mean tissue viability less than or equal to 50% indicates that the substance is an irritant, whereas a mean tissue viability greater than 50% indicates that the substance is a non-irritant.

### 2.7. Particle Size Determination

Particle size determinations were performed using a Zetasizer Pro Red (Malvern Panalytical, Malvern, UK). Two different test samples were prepared, one with one part 10% *w*/*v* HA and one part PBS, and the other with one part 10% *w*/*v* HA and one part 200 U/mL hyaluronidase. The particle size was measured immediately after mixing and then after one week using a material refractive index of 1.56 and absorption of 0.001 and a dispersant refractive index of 1.33 and viscosity of 1.02 mPa/s and duplicated 5 times. All tests were performed at 25 °C using a general-purpose analysis model and a 120 s equilibration time.

### 2.8. FTIR Spectroscopy

FTIR spectroscopy was performed using the Nicolet iS50 FT-IR (Thermo Scientific, Waltham, MA, USA). A hydrogel was prepared using the method from 2.2, and PBS was placed on top after gelling overnight. The gels were then placed at 37 °C for three weeks. The supernatant from the hydrogel and 10% *w*/*v* HA were analyzed using the iS50 ATR component, with 64 scans for each measurement in the MidIR region.

### 2.9. Statistical Analyses

Two-tail Student’s *t*-tests were used for two-group comparisons with α = 0.05. One-way ANOVA was used for comparisons with groups larger than two, using either Tukey’s multiple comparisons to compare all groups to one another or Dunnett’s multiple comparisons to compare all groups to a control group with α = 0.05. For the release profiles, either *t*-tests or one-way ANOVAs, depending on the number of groups, were used to evaluate whether the release each day differed between the gel types. For all figures, the error bars represent standard deviation values.

## 3. Results

### 3.1. Assessment of System Repeatability and Reproducibility

The thixotropic hydrogels consist of two main components: TEOS and HA, which are mixed and then adjusted to a pH of about 7.65 using NH_4_OH. To test the repeatability and reproducibility of the formulation process, we focused on assessing the effects of: (1) raw materials (2) pH range, and (3) HA concentration on the thixogels’ drug release properties, thixotropy, storage modulus, and dry substance.

**Figure 1:**
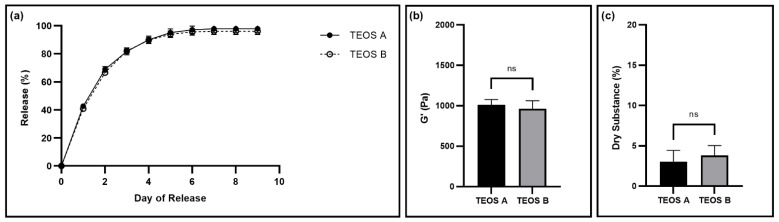
Evaluation of gels made with TEOS from two different manufactures, denoted TEOS A and TEOS B. PBS gels were used for all tests as to focus entirely on the effect of the TEOS. (**a**) The release profiles of fluorescein with N = 3. Error bars hidden by plot symbols when not visible. A *t*-test was used to compare release from the gels each day, indicating no statistically significant difference between the release profiles (Table S1). (**b**) Storage moduli for gels made using TEOS from two different manufacturers. Mean and standard deviation are displayed from data collected with N = 3. An unpaired *t*-test was used to compare the means, resulting in a *p*-value of 0.5430, with ns indicating no significant difference between the means. (**c**) Dry substance percentage for gels made using the two different manufacturers. Mean and standard deviation are displayed from data collected with N = 3. An unpaired *t*-test was used to compare the means, resulting in a *p*-value of 0.5069, with ns indicating no significant difference between the means.

First, we tested the gel properties of PBS-based gels made using TEOS from different manufacturers, denoted TEOS A and TEOS B. We used PBS-based gels rather than HA-based gels because we wanted to specifically focus on the effect of the TEOS. We performed release tests, using fluorescein as a model drug, on gels made using both manufacturers (Figure 1a). There was not a significant difference between the amount released any of the days (Table S1). Further, we tested the storage moduli of the gels, and there was not a statistically significant difference between the gels, highlighting that the TEOS type does not impact the gel mechanical properties (Figure 1b). Additionally, both gels continued to exhibit thixotropic behavior, and had no significant difference between dry substance (Figure S1 and Figure 1c).

**Figure 2:**
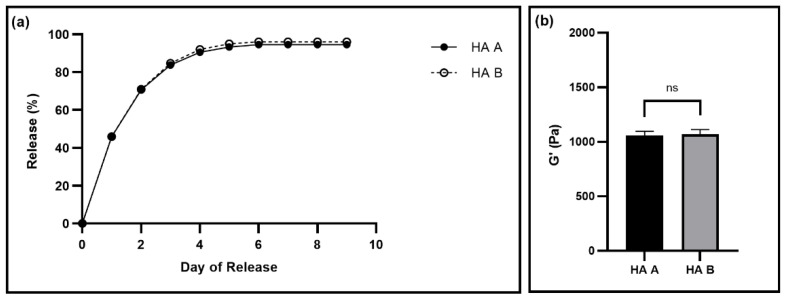
Evaluation of gels made with HA from two different manufactures, denoted HA A and HA B. (**a**) The release profiles of fluorescein with N = 3. Error bars hidden by plot symbols when not visible. A *t*-test was used to compare release from gels made using from HA the two different manufacturers each day, resulting in no significant *p*-values (Table S2). (**b**) Storage moduli for gels made from the two different HAs. Mean and standard deviation are displayed from data collected with N = 3. An unpaired *t*-test was used to compare the means, resulting in a *p*-value of 0.5656, with ns indicating no significant difference between the means.

Next, we tested whether the gel properties differed when using HA from two different manufacturers, denoted HA A and HA B, to ensure that the gel properties are not affected by different raw material sources. We performed fluorescein release and rheological tests on gels made with HA from the two different manufacturers (Figure 2). There was no statistically significant difference between the drug release or the storage moduli of the gels made using the different HA manufacturers (Figure 2). Additionally, both the gel types continued to exhibit thixotropic behavior (Figure S2).

**Figure 3:**
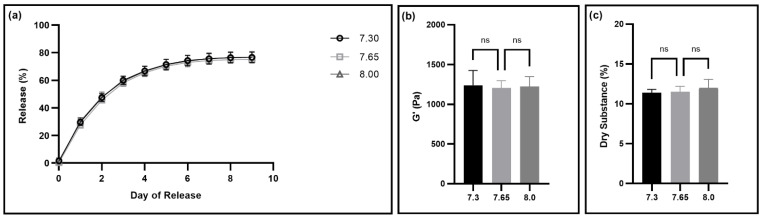
Evaluation of the impact of gel pH on gel properties. (**a**) The release profiles of fluorescein with N = 6. Error bars hidden by plot symbols when not visible. A one-way ANOVA was used to compare release from gels adjusted to different pH values, indicating no statistically significant difference between the release profiles (Table S3). (**b**) Storage moduli for gels made by adjusting to different pH values. Mean and standard deviation are displayed from data collected with N = 6. A one-way ANOVA was used to compare the means, with ns indicating no significant difference between the means. (**c**) Dry substance percentage for gels made by adjusting to different pH values. Mean and standard deviation are displayed from data collected with N = 6. A one-way ANOVA was used to compare the means, with ns indicating no significant difference between the means.

Next, we determined the range of pH values we could use to create gels with the desired properties. We tested the range from 7.3 to 8.0, testing gels adjusted to a pH of 7.3, 7.65, and 8.0 to determine whether the gels had the same properties for this range of pH values. For this, we also tested fluorescein release, storage modulus, thixotropy, and dry substance of the gels made by adjusting to different pH levels (Figure 3 and Figure S3). Our data indicate that there was no statistically significant difference between any of the gel properties for the gels made by adjusting to the three different pH levels (Figure 3).

**Figure 4:**
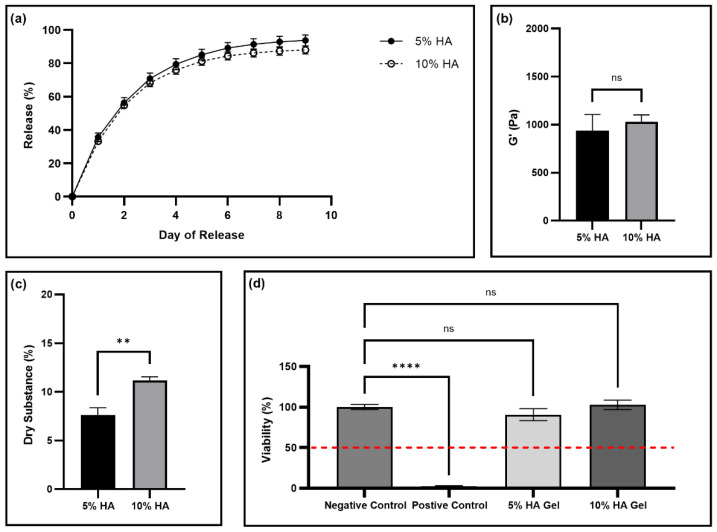
Evaluation of gels made with 5 and 10% *w*/*v* HA. (**a**) The release profiles of fluorescein with N = 3. Error bars hidden by plot symbols when not visible. A *t*-test was used to compare release from gels made from 5% versus 10% *w*/*v* HA each day, resulting no significant *p*-values (Table S4). (**b**) Storage moduli for gels made with different concentrations of HA. Mean and standard deviation are displayed from data collected with N = 3. An unpaired *t*-test was used to compare the means, resulting in a *p* = 0.5576, with ns indicating no significant difference between the means. (**c**) Dry substance percentage for gels made with different concentrations of HA. Mean and standard deviation are displayed from data collected with N = 3. An unpaired *t*-test was used to compare the means, resulting in a *p* = 0.0018, with ** indicating *p* < 0.01. (**d**) Skin relative viability compared to negative control tissue treatment of PBS to tissues treatment with positive control (5% SDS solution), 5% HA gel, and 10% HA gel, with N = 3 for the control groups and N = 6 for the treatment groups. The dashed line at the viability 50% indicates the cut off for a substance being a non-irritant, with all substances above the line being designated as a non-irritant. The treatments were compared to the negative control using a one-way ANOVA, with **** indicating *p* < 0.0001, and ns indicating not significant.

Finally, we sought to evaluate the effect of the reduction in the HA concentration in the gels, as a lower concentration would result in a significant decrease in production cost and ultimately lower product cost. For this, a 50% reduction in HA concentration was evaluated, corresponding to 5% *w*/*v* in the final formulation. We again tested fluorescein release, storage modulus, thixotropy, and dry substance of the gels made with the two different concentrations of HA (Figure 4a–c and Figure S4). There is not a significant difference between the fluorescein release or storage moduli of the gels made with the different percentages of HA (Figure 4a,b). However, as expected, there is a significant difference between the dry substance of the two gels (Figure 4c). To determine whether the gel made with a lower concentration of HA was a non-irritant, we performed a skin irritation test of gels made with 5% and 10% *w*/*v* HA (Figure 4d). All the treated tissues had mean viabilities greater than 50%, indicating they are non-irritants.

The obtained experimental data allowed us to establish overall thixogels’ manufacturing specification that would be used for subsequent quality control and shelf-life analyses. For the raw materials the specifications are as follows: TEOS purity ≤ 97.5% per manufacturer certificate of analysis, HA molecular weight 4.5 ± 0.5 kDa and polydispersity index 1.3 ± 0.05. For the thixogels, the set specifications are as follows: visual appearance clear to slightly opaque, storage modulus G’ 1080 ± 360 Pa, dry substance (DS) content 11.20 ± 0.72% for 10% *w*/*v* HA formulations and 7.62 ± 1.51% for 5% *w*/*v* HA formulations, respectively, pH 7.65 ± 0.35. Specifications were set as mean ± 2 standard deviations (mean ± 2 STD), except for the pH which was set as mean ± 1 standard deviations (mean ± 1 STD), considering the importance of this parameter on drug loading/release. In this study specifications were intentionally not set for drug release, as they would depend on the actual, application specific drug and the therapeutically desired release profile.

### 3.2. Release of Model Drugs

**Figure 5:**
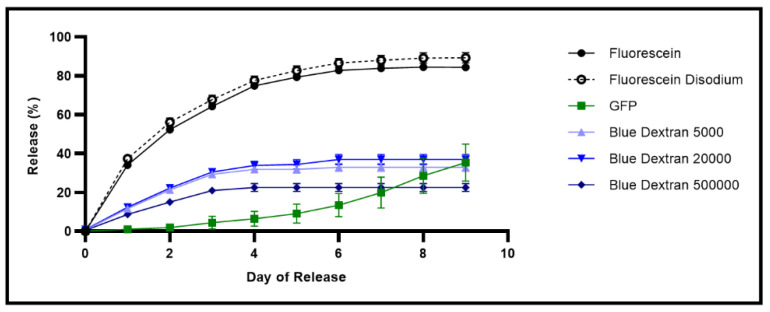
Release profiles of six different model drugs: fluorescein, fluorescein disodium, GFP, and blue dextran with molecular weights of 5000, 20,000, and 50,000 g/mol, from the hydrogels. A one-way ANOVA was used to compare release of the different model drugs each day, with no significant difference between the release of blue dextran with a molecular weight of 5000 and 20,000 g/mol any of the days, and no significant difference between the release of fluorescein and fluorescein disodium starting on day 3. All the other model drugs had significantly different release profiles until day 7, where the release for GFP and blue dextran 500,000 g/mol are not significantly different; day 8 where GFP release is not significantly different from any of the blue dextran release and blue dextran 5000 g/mol is not significantly different from 500,000 g/mol; and day 9 where GFP release is not significantly different from blue dextran with a molecular weight of 5000 and 20,000 g/mol, and blue dextran 5000 g/mol is not significantly different from 500,000 g/mol (Table S5).

To test whether the gels would be applicable for drug release after manufacturing, we tested the release of various model drugs from the gels. We tested release of fluorescein, fluorescein disodium, GFP, and blue dextran at molecular weights of 5000, 20,000, and 500,000 g/mol over the course of 9 days (Figure 5). There was not a significant difference between the release of blue dextran with a molecular weight of 5000 g/mol and 20,000 g/mol for any of the days (Table S5). Additionally, there was no significant difference between the release of fluorescein and fluorescein disodium from day 3 until the end of the release study (Table S5). GFP had no significant difference in compared blue dextran with a molecular weight of 500,000 g/mol for day 7 and 8 and compared to blue dextran with a molecular weight of 5000 and 20,000 for days 8 and 9 (Table S5). Blue dextran with a molecular weight of 5000 and 500,000 g/mol had no significant differences days 8 and 9 (Table S5). All the other drugs had significant differences between the total drug released each day (Table S5).

**Figure 6:**
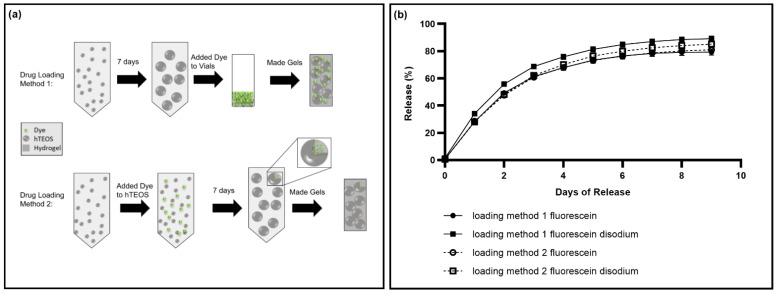
Evaluation of drug release using different drug loading methods. (**a**) The two different drug loading methods. In method 1, the hTEOS was made, then let sit for 7 days. After 7 days, gels were made in glass vials containing the dye, either fluorescein or fluorescein disodium. In method 2, the hTEOS was made, then then the dye was added and then let sit for 7 days, after which point the gels were made. (**b**) The release profiles of fluorescein and fluorescein disodium from gels using the two different drug loading methods from (**a**), with N = 3. Error bars are hidden by symbols when not visible. A *t*-test was used to compare release using method 1 versus method 2 for fluorescein and fluorescein disodium. There was no statistically significant difference between the release profiles using method 1 and 2 for fluorescein (Table S6). There was a statistically significant difference between the release of fluorescein disodium loaded using method 1 versus 2.

In addition to testing the release of various model drugs, we also wanted to evaluate different drug loading strategies to see how they would impact the release profiles. In the first loading strategy, the hTEOS was incubated at room temperature for 7 days prior to making gels in the usual way, to entrap the drugs in the hydrogel network (Figure 6a). In the second loading strategy, the hTEOS was incubated at room temperature with the drug for seven days prior to making the gels, with the goal of encapsulating the drug within the hTEOS nanoparticles (Figure 6a). There was no significant difference between the release profiles for fluorescein (Table S6). In contrast, there was a statistically significant difference between the release of fluorescein disodium using the two different loading strategies, with fluorescein disodium being released at a slower rate using loading strategy 2 compared to strategy 1 (Table S6).

### 3.3. Impact of Ear-Specific Physiological Factors on Release

**Figure 7:**
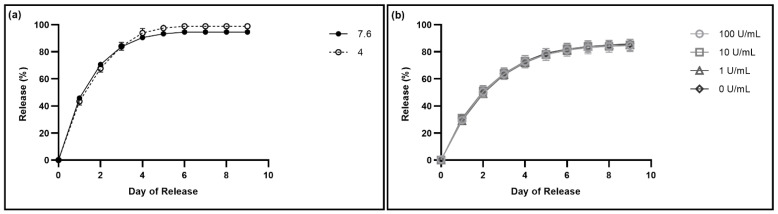
Impact of physiological factors on drug release from the thixogels. (**a**) Release of fluorescein into PBS with different pH values (7.6 and 4.0), with N = 3 for each release profile. Error bars hidden by plot symbols when not visible. A *t*-test was used to compare release into PBS with a pH of 7.6 versus 4.0 each day, indicating no statistically significant difference between the release profiles (Table S7). (**b**) Release of fluorescein into PBS with different concentrations of hyaluronidase (0, 1, 10, and 100 U/mL), with N = 6 for 100 U/mL and N = 3 for the other release profiles. Error bars hidden by plot symbols when not visible. A one-way ANOVA was used to compare release each day, indicating no statistically significant difference between the release profiles (Table S8).

There are two main factors in the outer ear that may impact the release of drugs from these hydrogels: ear pH and the presence of factors/enzymes that depolymerize HA. The pH of the ear is usually acidic, and becomes more neutral when the ear is infected, ranging from 7.1 to 7.8 [25,26]. As a result, we wanted to investigate the impact of pH on the drug release from the gels. We studied the release of fluorescein from gels into PBS with a pH of 4.0 versus 7.6, with 4.0 representing a healthy ear, and 7.6 representing the pH of an infected ear (Figure 7a). There was no statistically significant difference between the drug release into the different pH levels (Table S7).

The other outer ear canal-specific factor to be considered regarding drug release from these hydrogels was presence of factors/enzymes that depolymerize HA which we modeled using hyaluronidase, an enzyme that specifically degrades HA ubiquitous in the human body, and which would represent the most accelerated degradation profile of the glycosaminoglycan [27,28]. As HA is one of the major components of the thixogels, we sought to test the effects of HA depolymerization on the gels’ drug release profiles. No significant difference was detected between the release profiles of samples treated with various hyaluronidase concentrations compared to the release into PBS without hyaluronidase (Table S8). To test the efficacy of the hyaluronidase on this HA at a pH of 7.6, we compared the particle size of HA in a 10% *w*/*v* solution after a weeklong incubation with hyaluronidase to the particle size of HA in a 10% *w*/*v* solution after a weeklong incubation in PBS, and found there was a significant decrease in particle size with the presence of hyaluronidase (Figure S5). To test whether HA was being released by the gels into the PBS, we compared the FTIR profiles of HA to the supernatant of a hydrogel with PBS on top, and found they have the same peaks, indicating that the HA is being released by the gels (Figure S6).

## 4. Discussion

The goal of this work was to determine the manufacturability of our previously described tetraethyl orthosilicate-based thixogels [16]. For this, we investigated three major facets of the design and manufacturing process of these gels: the effect of raw materials sources on gel properties, the loading and release of model drugs with different properties, and the impact of target application-specific physiological factors on drug release profiles. First, we completed tests to ensure that the thixogels had consistent, within specifications properties regardless of the suppliers of TEOS or HA. These comparisons were made to ensure supply chain independence. Thixogels made using the two different sources of TEOS, had no significant difference between storage moduli, drug release, or dry substance, indicating manufacturing process and supply chain robustness (Figure 1). The storage moduli and drug release from thixogels made using HA from different manufacturers were also within specifications and not statistically different from each other (Figure 2). Additionally, all the thixogels continued to display thixotropic behavior (Figures S1 and S2). These results indicate that the provenance of TEOS and HA does not have a significant impact on the gel properties, leading to a repeatable and reproducible manufacturing process and a robust multiple sourcing supply chain, as the TEOS and HA can be procured from a multitude of manufacturers.

Next, we determined a tolerance range for gel pH adjustment. To induce gelation, the pH value needs to be around 7.65. To simplify the manufacturing process, we wanted to determine the range of pH values that results in thixogels with the consistent, within specification properties. Gels made with pH levels of 7.3, 7.65, and 8.0 showed consistent properties, indicating that there is a wide tolerance range for pH adjustment during manufacturing (Figure 3). This simplifies the manufacturing process and allows for less rigorous control over exact pH values. A wide range of acceptable pH levels will speed up the manufacturing process, with less time spent trying to achieve an exact pH value, and thus increase the amount of time where the thixogels are still in solution, again simplifying the manufacturing process and prolonging the timing between subsequent handling steps.

Further, we evaluated the effect of the reduction in the concentration of HA in the thixogels, as HA is the most expensive component; therefore, reducing the amount of HA would significantly reduce the cost of the manufacturing process and ultimately of the final product. Gels made with 5% *w*/*v* HA had the same release profile and storage moduli as the usual 10% *w*/*v* HA gels (Figure 4a,b). Additionally, the 5% *w*/*v* HA gels continued to exhibit thixotropic behavior (Figure S4). As expected, the dry substance of the 5% *w*/*v* HA gel is significantly smaller than the 10% *w*/*v* HA gel (Figure 4c). This result is intuitive, as the 10% HA gel has twice the amount of HA, corresponding to more dry material. The difference in dry substance does not appear to impact any other gel properties. Further, neither gel type was a skin irritant, with no significant difference between the gel treatments and the control PBS treatment, and both satisfying the standardized skin irritation assay acceptance criteria with treatments having viabilities significantly greater than 50% (Figure 4d). As a result, the 5% *w*/*v* gel formulation appears a suitable product alternative, as it has the same properties as the 10% *w*/*v* gel but would have significantly lower cost of production.

As the desired application of this hydrogel is antibiotic delivery for treatment of otitis externa, we next assessed the release of various model drugs from the thixogels. For this, model drugs with a wide range of properties were selected: small molecules with different lipophilicity/hydrophilicity index values (fluorescein, fluorescein disodium), polymers of different molecular weights (blue dextran at molecular weights of 5000, 20,000, and 500,000 g/mol), and a protein (GFP) (Figure 5). Blue dextran of 5000 g/mol showed no statistically significant differences in the release profile compared to that of 20,000 g/mol blue dextran (Table S5). However, blue dextran with a molecular weight of 500,000 g/mol was significantly different from 20,000 g/mol for the entirety of release, and different from 5000 g/mol up until day 8 (Table S5). This behavior is most likely related to the polymers’ molecular weight differences, with the larger molecular weight being more entrapped by the thixogels’ network compared to the smaller molecular weight blue dextrans. The similarity between the release of 20,000 and 5000 g/mol appears to indicate that there is a size cutoff between 500,000 and 20,000 g/mol that causes higher entrapment in the hydrogel network. Additionally, all the blue dextran samples stopped releasing at day 3 or 4 of release (Figure 5), prior to releasing the entire loaded amount, indicating that a portion of the polymer is permanently retained by the hydrogel network and not released, not a surprising result as dextran is often used as a interpenetrating polymer network in hydrogels [29,30,31]. Along with the blue dextran, the two small molecules, fluorescein and fluorescein disodium, also had very similar release profiles, only differing for the first two days of release (Table S5). The difference for the first two days could be due to several factors. First, fluorescein is smaller than fluorescein disodium, with molecular weights of 332.3 g/mol and 376.3 g/mol, respectively [32,33]. The difference in molecular weight could make fluorescein more easily encapsulated by the nanoparticles in the hydrogel network, and thus released more slowly initially. Alternatively, fluorescein and fluorescein disodium also have distinct logP values, with fluorescein being more hydrophobic with a logP of 3.35 versus −0.67 for fluorescein disodium [32,33]. As a result, fluorescein disodium may be more readily released into the PBS and therefore released more quickly initially. The different logP values could also make fluorescein more easily encapsulated by the nanoparticles, which would also contribute to the slower release of fluorescein at the start of the release. GFP had a distinct release profile compared to the other model drugs, likely due to non-specific interactions between the protein and thixogel components, along with the large size of the GFP, leading to a slower release. Overall, these results indicate that the thixogels can be loaded with and release a wide variety of drugs with distinct release profiles and highlight the versatility of this drug delivery system. Additionally, these findings indicate that these materials may be used for incorporating two or more different drugs to obtain tailored release patters, which may broaden the applicability of these materials.

Next, we assessed the impact of different drug loading strategies on drug release profiles. Previously, we found that the hTEOS nanoparticle size increases over time [20]. As a result, we wanted to investigate how this increasing nanoparticle size would impact the drug release. We used two different loading strategies (Figure 6a), where the goal of the first loading strategy was to entrap the drug in the polymer network, and the goal of the second was to encapsulate the drug in the hTEOS nanoparticles. There was not a significant difference between the fluorescein release profiles using the two different loading strategies (Table S6), which may be due to several different reasons. First, fluorescein may be encapsulated by the nanoparticles using both loading strategy 1 and 2 due to its high hydrophobicity, causing negligible differences between the release profiles [32]. Alternatively, fluorescein may quickly adsorb to the surfaces of the nanoparticles in both cases, leading to similar release profiles. In contrast, fluorescein disodium was released significantly slower using method 2 versus method 1 (Table S6). Fluorescein disodium is less hydrophobic than fluorescein, meaning it would likely be encapsulated by the nanoparticles more slowly, and therefore would be much more encapsulated using loading strategy 2 compared to 1, causing the slower release [32,33]. These results indicate that the drug release profiles may be altered by using alternative loading strategies for some drugs, again broadening the applicability of these thixogels and highlighting this drug delivery system’s versatility.

Finally, we investigated the impact of two application specific physiological factors, specifically local pH and enzymatic activity, on drug release from the hydrogels. The pH of the ear varies widely, with a healthy ear typically having an acidic pH, and an infected ear having a pH from 7.1 to 7.8 [25,26]. The drug release from the hydrogels into PBS with a pH of 7.6 was not significantly different from the release into PBS with a pH of 4.0, indicating that patient ear pH would not impact the efficacy of the treatment (Table S7). Additionally, there is no significant difference between the release of fluorescein in the presence of the various concentrations of hyaluronidase (Table S8), indicating that the depolymerization of the thixogel-constituent HA is not significantly impacting the release of the loaded drug; this is a somewhat surprising result, as the release of fluorescein from gels without HA is significantly different from release from 10% *w*/*v* HA gels for the first day (Figure S7). One explanation for this observation is that the drug containment process is primarily dictated by the thixogel constituent TEOS nanoparticles with a much lower impact from the HA component of the thixogel, most likely arising from non-specific physical interactions between the HA polymer chains and releasing drugs withing the initial release times as observed in Figure S7. Dynamic light scattering results indicate that the HA particle sizes are significantly reduced by the hyaluronidase (Figure S5), suggesting that the enzymatic HA depolymerization is indeed happening and that the observed release profiles in the presence of hyaluronidase are not due to impaired enzymatic activity. However, an alternative explanation is that the presence of HA in the gel formation step does impact the drug release as observed in Figure S7, but that the HA itself is rapidly released from the gel as well, causing the presence of hyaluronidase to not have an impact on the drug release profile. Our FTIR analyses seem to indicate this to be the case, with the thixogel supernatant having the same profile as a 10% *w*/*v* HA solution. This would account for the lack of difference between the release profiles at different concentrations of hyaluronidase, as the hyaluronidase would simply be degrading the released HA from the supernatant rather than the drug-loaded thixogel (Figure S6). These are promising results, as they indicate that the outer ear canal-specific physiological factors likely would not impact the drug release from the hydrogels, making these drug delivery systems highly predictable in their behavior for therapeutic applications.

In summary, this study highlights the manufacturability of tetraethyl orthosilicate-based hydrogels for various drug release application. Our data show that the thixogels have a robust and reproducible manufacturing process, independent of the supply chain, and have a wide tolerance for pH adjustment, making the manufacturing process more flexible. Additionally, we found that the gels can be formulated with a lower concentration of HA, thus reducing the cost of the production and ultimately of the product. Further, the thixogels can incorporate and release a wide variety of model drugs, including small molecules, polymers, and proteins; and different loading strategies can be used to obtain tailored, application-specific release profiles. Lastly, we have showed that the loaded drug release profiles are not impacted by outer ear canal-specific pH or potential HA-specific depolymerization. Overall, these results highlight yet again the versatility of this drug release system, its readiness for manufacturing, overall product development and subsequent advancement to market.

## Data Availability

The data presented in this study are available on request from the corresponding author.

## Supplementary Materials

The following supporting information can be downloaded at: https://www.mdpi.com/article/10.3390/pharmaceutics14102020/s1, Figure S1: Thixotropic profiles of gels obtained with TEOS sourced from different manufactures; Figure S2: Thixotropic profiles of gels obtained with HA sourced from different manufactures; Figure S3: Thixotropic profiles of gels prepared at various pH levels; Figure S4: Thixotropic profiles of gels prepared with HA at 5% *w*/*v* and 10% *w*/*v*; Figure S5: Average HA particle size after one week for HA alone and HA with hyaluronidase; Figure S6: FTIR absorbance of 10% *w*/*v* HA thixogel supernatant and control HA 10% *w*/*v* solution; Figure S7: The release profiles of fluorescein from gels made with PBS and 10% *w*/*v* HA; Table S1: *p*-values from independent t-tests comparing daily total drug release from thixogels made using TEOS from different manufactures; Table S2: *p*-values from independent t-tests comparing daily total drug release from thixogels made using HA from different manufactures; Table S3: *p*-values from one-way ANOVAs, using Dunnett’s correction for multiple comparisons versus control (release from gel prepared at a pH of 7.65) of total daily drug release from thixogels adjusted to various pH values; Table S4: *p*-values from independent t-tests comparing daily total drug release from thixogels prepared with 5% *w*/*v* and 10% *w*/*v* HA; Table S5: *p*-values from one-way ANOVAs, using Tukey’s correction for multiple comparisons of daily total release of fluorescein, fluorescein disodium, green fluorescent protein and blue dextran with molecular weights of 5000, 20,000, and 500,000 g/mol; Table S6: *p*-values from independent t-tests comparing daily total drug release from thixogels using the two different loading methods for fluorescein and fluorescein disodium; Table S7: *p*-values from independent t-tests comparing daily total drug release from thixogels into pH of 4.0 versus 7.6; Table S8: *p*-values from one-way ANOVAs, using Dunnett’s correction for multiple comparisons versus control (0 U/mL hyaluronidase) of daily total release into different levels of hyaluronidase.
