# Supplementary material for: Manufacturability of a Tetraethyl Orthosilicate-Based Hydrogel for Use as a Single Application Otitis Externa Therapeutic
Source: Pharmaceutics. 2022 Sep 23;14(10):2020. doi: 10.3390/pharmaceutics14102020 (PMC9607239; doi:10.3390/pharmaceutics14102020)
Supplement: Supplementary file 1 [file pharmaceutics-14-02020-s001.zip › pharmaceutics-1909956-supplementary.pdf]

Supplementary Materials for Manufacturability of a Tetraethyl Orthosilicate-Based Hydrogel for Use as a Single Application Otitis Externa Therapeutic

Table S1. P-values from independent t-tests comparing daily total drug release from thixogels made using TEOS from different manufactures with N=3.

Day of Release	*p-value*
1	0.1530
2	0.1557
3	0.8761
4	0.8514
5	0.6125
6	0.4937
7	0.4349
8	0.4349
9	0.4349

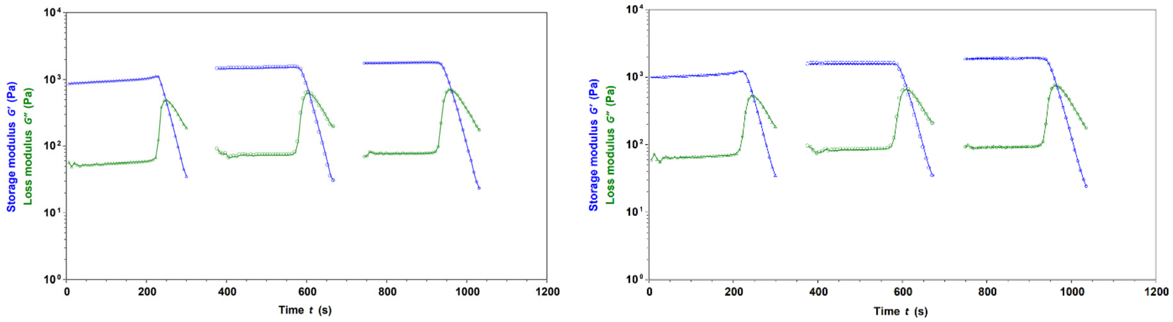

Figure S1. Thixotropic profiles of gels obtained with TEOS sourced from different manufactures, with TEOS A on the left and TEOS B on the right.

Table S2. P-values from independent t-tests comparing daily total drug release from thixogels made using HA from different manufactures with N=3.

Day of Release	*p-value*
1	0.6626
2	0.5405
3	0.3168
4	0.1406
5	0.1116
6	0.1511
7	0.1557
8	0.1557
9	0.1557

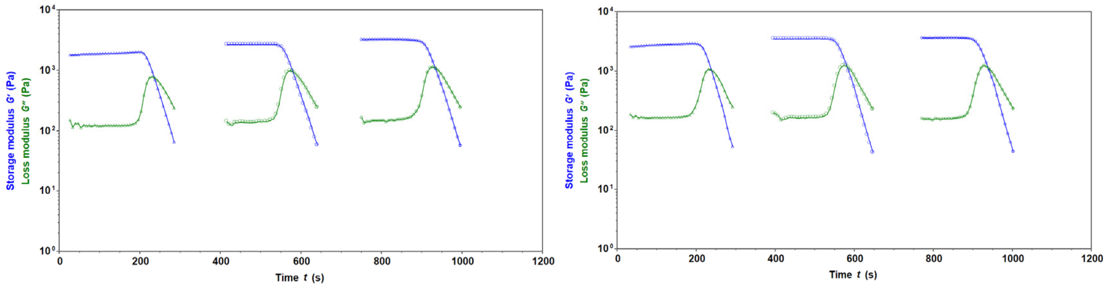

Figure S2. Thixotropic profiles of gels obtained with HA sourced from different manufactures, with HA A on the left and HA B on the right.

Table S3. P-values from one-way ANOVAs, using Dunnett's correction for multiple comparisons versus control (release from gel prepared at a pH of 7.65, N=6) of total daily drug release from thixogels adjusted to various pH values.

Day of Release	*Overall p-value*	*8.00 p-value*	*7.30 p-value*
1	0.3308	0.2355	0.3734
2	0.5607	0.4464	0.5681
3	0.6808	0.8037	0.5643
4	0.8422	0.8431	0.7654
5	0.8799	0.8702	0.8200
6	0.8669	0.8791	0.7961
7	0.8558	0.8051	0.8155
8	0.8154	0.7601	0.7635
9	0.8129	0.7474	0.7720

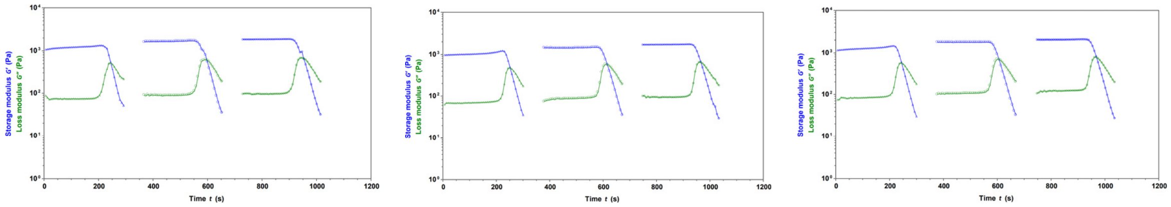

Figure S3. Thixotropic profiles of gels prepared at pH to 7.30 (left), 7.65 (middle), and 8.00 (right).

Table S4. P-values from independent t-tests comparing daily total drug release from thixogels prepared with 5% w/v and 10% w/v, respectively HA with N=3.

Day of Release	*p-value*
1	0.1796
2	0.4603
3	0.3431
4	0.2195
5	0.1769
6	0.1149
7	0.0949
8	0.0863
9	0.0749

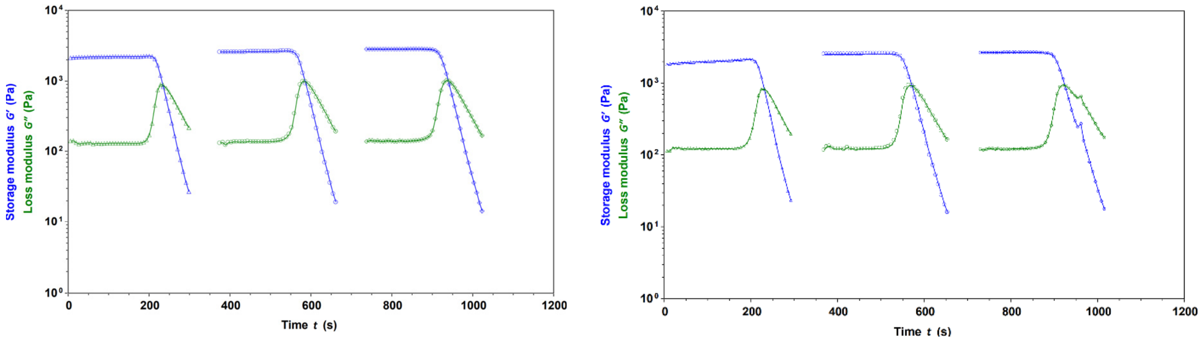

Figure S4. Thixotropic profiles of gels prepared with HA at 5% *w/v* and 10% *w/v*, respectively, with 5% on the left and 10% on the right.

Table S5. P-values from one-way ANOVAs, using Tukey's correction for multiple comparisons of daily total release of fluorescein (Fl), fluorescein disodium (FlNa), green fluorescent protein (GFP) and blue dextran with molecular weights of 5,000 (B5), 20,000 (B20), and 500,000 (B500) g/mol with N=3.

Day of Release	*Overall p-value*	*Fl vs. FlNa*	*Fl vs. GFP*	*Fl vs. B5*	*Fl vs. B20*	*Fl vs. B500*	*FlNa vs. GFP*	*FlNa vs. B5*	*FlNa vs. B20*	*FlNa vs. B500*	*GFP vs. B5*	*GFP vs. B20*	*GFP vs. B500*	*B5 vs. B20*	*B5 vs. B500*	*B20 vs. B500*
1	<0.0001	0.0027	<0.0001	<0.0001	<0.0001	<0.0001	<0.0001	<0.0001	<0.0001	<0.0001	<0.0001	<0.0001	<0.0001	0.8514	0.0073	0.0011
2	<0.0001	0.0319	<0.0001	<0.0001	<0.0001	<0.0001	<0.0001	<0.0001	<0.0001	<0.0001	<0.0001	<0.0001	<0.0001	0.9325	0.0007	0.0002
3	<0.0001	0.3248	<0.0001	<0.0001	<0.0001	<0.0001	<0.0001	<0.0001	<0.0001	<0.0001	<0.0001	<0.0001	<0.0001	0.9732	0.0029	0.0009
4	<0.0001	0.7259	<0.0001	<0.0001	<0.0001	<0.0001	<0.0001	<0.0001	<0.0001	<0.0001	<0.0001	<0.0001	<0.0001	0.8929	0.0043	0.0008
5	<0.0001	0.6622	<0.0001	<0.0001	<0.0001	<0.0001	<0.0001	<0.0001	<0.0001	<0.0001	<0.0001	<0.0001	0.0006	0.8576	0.0122	0.0019
6	<0.0001	0.6749	<0.0001	<0.0001	<0.0001	<0.0001	<0.0001	<0.0001	<0.0001	<0.0001	<0.0001	<0.0001	0.0338	0.6287	0.0142	0.0011
7	<0.0001	0.7692	<0.0001	<0.0001	<0.0001	<0.0001	<0.0001	<0.0001	<0.0001	<0.0001	0.0114	0.0014	0.9459	0.7860	0.0498	0.0057
8	<0.0001	0.7518	<0.0001	<0.0001	<0.0001	<0.0001	<0.0001	<0.0001	<0.0001	<0.0001	0.7809	0.2077	0.5228	0.8418	0.0825	0.0115
9	<0.0001	0.7520	<0.0001	<0.0001	<0.0001	<0.0001	<0.0001	<0.0001	<0.0001	<0.0001	0.9806	0.9976	0.0339	0.8666	0.1053	0.0164

Table S6. P-values from independent t-tests comparing daily total drug release from thixogels using the two different loading methods for fluorescein and fluorescein disodium with N=3.

Day of Release	*Fluorescein p-value*	*Fluorescein Disodium p-value*
1	0.6476	0.0114
2	0.2446	0.0061
3	0.8085	0.0130
4	0.9028	0.0226
5	0.9161	0.0374
6	0.9480	0.0377
7	0.8414	0.0446
8	0.5166	0.0508
9	0.2903	0.0625

Table S7. P-values from independent t-tests comparing daily total drug release from thixogels into pH of 4.0 versus 7.6 with N=3.

Day of Release	*p-value*
1	0.2001
2	0.1616
3	0.8748
4	0.1665
5	0.1035
6	0.1056
7	0.1061
8	0.1061
9	0.1061

Table S8. P-values from one-way ANOVAs, using Dunnett's correction for multiple comparisons versus control (0 U/mL hyaluronidase) of daily total release into different levels of hyaluronidase. N=6 for 100 U/mL and N=3 for all other samples.

Day of Release	*Overall p-value*	*100 U/mL p-value*	*10 U/mL p-value*	*1 U/mL p-value*
1	0.4814	0.5881	>0.9999	0.4817
2	0.8910	0.9927	0.9642	0.9642
3	0.9826	0.9944	>0.9999	0.9703
4	0.9850	0.9951	>0.9999	0.9743
5	0.9824	0.9641	0.9749	0.9749
6	0.9793	0.9826	>0.9999	0.9752
7	0.9576	0.9508	>0.9999	>0.9999
8	0.9713	0.9678	0.9757	>0.9999
9	0.9581	0.8961	0.9758	0.9758

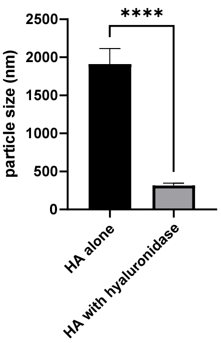

Figure S5. Average particle size after one week for HA and HA with hyaluronidase, with N=5 and **** indicating p<0.0001.

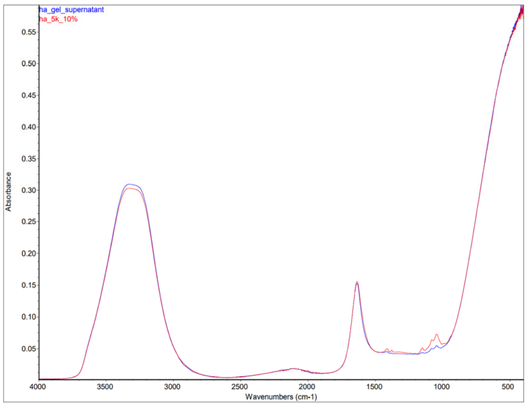

Figure S6. FTIR absorbance of 10% w/v HA thixogel supernatant (blue) and control HA 10% w/v solution (red).

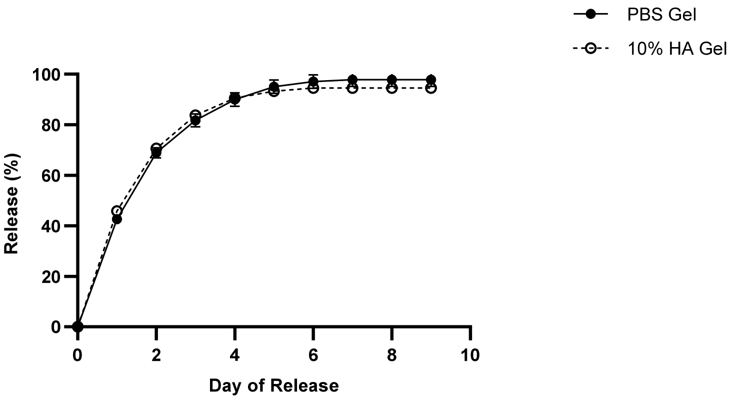

Figure S7. The release profiles of fluorescein from gels made with PBS and 10% HA, with N=3 for each release profile. Error bars hidden by plot symbols when not visible. A t-test was used to compare release from gels made each day, resulting in a statistically significant p value for the first day of release (p=0.0279).
